# Seal bites at sub-Antarctic Marion Island: Incidence, outcomes and treatment recommendations

**DOI:** 10.4102/jsava.v91i0.1720

**Published:** 2020-03-24

**Authors:** Ryan R. Reisinger, Miles Penfold, Marthán N. Bester, Gerhard Steenkamp

**Affiliations:** 1 Department of Zoology and Entomology, Mammal Research Institute, University of Pretoria, Pretoria, South Africa; 2 Department of Zoology, Nelson Mandela University, Port Elizabeth, South Africa; 3 Department of Companion Animal Clinical Studies, Faculty of Veterinary Science, University of Pretoria, Pretoria, South Africa

**Keywords:** marine mammal, bite, treatment, infection, zoonoses

## Abstract

Seal biologists at Marion Island (Southern Ocean) are in frequent contact with seals. During research activities, biologists may be bitten by seals, yet no standardised protocol for treating such bites is in place. Information on 22 seal bite cases at Marion Island was collected. Treatment of these bites varied, reflecting a need for standardised protocols for the treatment of bites. Recommendations for the in-field treatment of bites are presented. Five of the 22 cases had some symptoms which resembled ‘seal finger’ – a zoonotic infection, usually of the hands, that is contracted after a person comes into contact with tissues of seals or is bitten by one. However, in four of these cases, symptoms subsided within 4 days without antibiotic treatment; in the fifth case antibiotics were administered and symptoms subsided in 4 days. There is little evidence of the occurrence of seal finger at Marion Island, but this deserves further investigation.

## Introduction

The Prince Edward Islands (46°54′S, 37°45′E) – comprising Marion Island and the smaller Prince Edward Island – are situated in the southern Indian Ocean. The islands support multiple breeding populations of three seal species: southern elephant seals *Mirounga leonina* (Phocidae), sub-Antarctic fur seals *Arctocephalus tropicalis* (Otariidae) and Antarctic fur seals *Arctocephalus gazella* (Otariidae) (Bester et al. 2011). Leopard seals *Hydrurga leptonyx* (Phocidae) are frequent transients during the austral spring and summer (Bester et al. 2006). Marine mammal research has been conducted on the islands since 1951 and a formal research programme commenced in 1973. Thenceforth, one to five marine mammalogists and their assistants were deployed annually on Marion Island (see Bester et al. 2011 for a history of the programme). During their research and monitoring activities, the seal biologists come in frequent contact with seals. For example, from 1993 to 2014, approximately 15 000 sub-Antarctic fur seal pups have been weighed (Oosthuizen et al. 2016) and seal biologists regularly draw blood from fur seals. During these activities, biologists may be bitten by seals, yet no standardised protocol for treating such bites is in place. Furthermore, seal biologists handle seal carcasses to perform necropsies or for osteological collections, and collect and process seal faeces. Protective measures, such as wearing gloves, are seldom undertaken (R.R. Reisinger and M.N. Bester, pers. obs.). It is therefore interesting that despite this there have been no reports of the zoonotic infection ‘seal finger’ from the island. This infection of the hands is contracted after a person comes into contact with blood, blubber or other tissues of pinnipeds or is bitten by one (Tryland 2018). The disease is common amongst professional seal hunters and those handling seal pelts and carcasses and, increasingly, amongst other professionals handling seals (biologists, aquarium personnel, wildlife workers, etc.) (Hartley & Pitcher 2002; Hunt et al. 2008). Most reports are from the northern hemisphere (e.g. Candolin 1953; Rodahl 1952; Waage 1950); in the southern hemisphere, only a small number of hand infections with clinical presentation similar to seal finger have been reported (Cawthorn 1994; Liavaag 1940; Panagis, Apps & Knight 1982).

The aim of this research was, firstly, to document the seal bites that researchers sustained at Marion Island over a 17-year period (1995–2012). Secondly, the treatment these researchers received was recorded and related to outcome. Thirdly, we offer suggestions for the treatment of seal bite wounds. Lastly, we discussed the apparently low risk of contracting ‘seal finger’ at this location.

## Materials and methods

We contacted Marion Island seal biologists and asked them to provide details of any seal bites they had sustained, including treatment of the bite (as well as antibiotics administered, if any) and symptoms associated with the bite in the bitten limb. The South African Department of Environmental Affairs (DEA) released their available medical records for the island (*n* = 3 bite wounds) and medical staff (*n* = 3) that were stationed on the island and provided their personal records or recollections of bites.

### Ethical considerations

Seal research on Marion Island was permitted by the Prince Edward Islands Management Committee (C06-08) and approved by the University of Pretoria’s Animal Use and Care Committee (AUCC 040827-024).

## Results

### Bite responses

**FIGURE 1:**
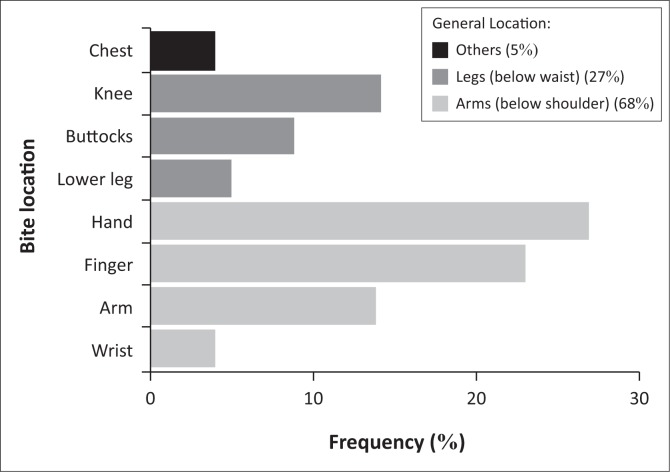
Anatomical distribution of 22 seal bites sustained by field biologists at Marion Island from 1995 to 2012.

Eight field biologists (six men and two women, aged 23–28 years) reported 22 cases of seal bites from 1995 to 2012 (Table 1); three of these were included in the medical records. Biologists were bitten by all three of the seal species breeding on the island, but never by leopard seals. Sub-Antarctic fur seals were responsible for most of the bites (12 bites), followed by Antarctic fur seals (seven bites). Southern elephant seals were responsible for only three bites. Most bites (11) were sustained on the hands and fingers, but bites were also sustained on the forearms, knees, lower leg, buttocks, wrist and chest (Table 1; Figure 1). Treatment of bite wounds ranged from no treatment at all to thorough scrubbing with antiseptics, debridement, suturing and administering antibiotics (tetracycline and amoxicillin + clavulanic acid). Five cases (Table 1; cases 5–8 and 13) had some symptoms that could possibly resemble seal finger (Hartley & Pitcher 2002; Rodahl 1952; Tryland 2018), but in four of these cases, symptoms subsided within 4 days, without antibiotic treatment. In the fifth case (Table 1; case 13), antibiotics (including tetracycline) were administered and symptoms subsided in 4 days.

**TABLE 1:** Summary of seal bites suffered by field biologists at sub-Antarctic Marion Island, 2005–2012.

Case	Patient	Seal			bite						
		Species	Age	Sex	Description	Location	Treatment	Antibiotics	Symptoms	Seal finger symptoms	Persistence†
1	A	ML	Adult	F	3-cm laceration, multiple shallow punctures	Left arm	Bathed in Betadine, flushed with saline – multiple times (three to eight) with syringe. Cleaned with Betadine over next few days. Anti-tetanus	Yes; unknown	-	No	NA
2	A	AG	Juvenile	U	Puncture	Left buttock	Wiped with Betadine	No	-	No	NA
3	A	AG	Pup	U	Various skin breaks	Hands	Cleaned with ethanol	No	Slight redness	No	NA
4	A	AT	Pup	U	Various skin breaks	Hands	Cleaned with ethanol	No	Slight redness	No	NA
5	B	AT	Pup	U	Lacerations	Fingers (three, four)	Washed with soap	No	Severe swelling, stiffness and infection	Yes	4 days, treated with peroxide, then immediately recovered
6	B	AT	Pup	U	Lacerations	Knee	None	No	Slight swelling	?	1 day, then recovered without treatment
7	B	AT	Pup	U	Puncture and laceration	Finger (one)	Washed with medical handwash	No	Slight swelling, purulent discharge	?	2 days, then recovered without treatment
8	B	AT	Pup	U	Laceration	Forearm	None	No	Slight swelling around laceration	?	2 days, then recovered without treatment
9	B	AG	Pup	U	Various lacerations and punctures	Hands		No		No	NA
10	B	AT	Pup	U	Various lacerations and punctures	Hands		No		No	NA
11	C	ML	Adult	F	Long laceration	Wrist	Anti-tetanus, sutures	Yes; unknown		No	NA
12	C	AT	Juvenile	U	Multiple punctures	Chest	Anti-tetanus, sutures	Yes; unknown		No	NA
13	D	AT	Adult	M	Puncture and severe lacerations	Knee	Cleaned with Betadine, sutures, cleaned with Betadine twice daily for 2 weeks	Yes; unknown and tetracycline	Swelling and stiffness	Yes	3–4 days
14	E	AG	Juvenile	M	Two punctures	Lower leg	Flushed with Savlon	No		No	NA
15	F	AG	Pup	U	Various small lacerations	Forearms	Scrubbed with saline and Savlon	No		No	NA
16	F	AT	Pup	U	Various small lacerations	Knees	Scrubbed with saline and Savlon	No		No	NA
17	G	AT	Adult	F	3-cm laceration	Finger (two)	Applied Betadine	No		No	NA
18	G	AG	Adult	F	Deep puncture wound	Finger (three)	Rinsed with saline, anti-tetanus	Yes, tetracycline		No	NA
19	G	AG	Pup	U	Various lacerations and punctures	Hands	Cleaned with Hibitane or ignored	No		No	NA
20	G	AT	Pup	U	Various lacerations and punctures	Hands	Cleaned with Hibitane or ignored	No		No	NA
21	G	AT	Adult	F	Avulsion	Finger (one)	Scrubbing, debridement	Yes, tab Co-amoxiclav, 5 days		No	NA
22	H	ML	Adult	F	Puncture	Buttock	Cleaned wound, some debridement	Yes, tab Co-amoxiclav, 5 days		No	NA

Species: ML, *Mirounga leonina*; AG, *Arctocephalus gazella*; AT, *Arctocephalus tropicalis*.

Sex: F, female; M, male; U, unknown.

Patients: A – male, 24 years; B – male, 24–28 years; C – male; D – female, 23 years; E – female, 28 years; F – male 26–27 years; G – male, 23–27 years; H – male, 23 years. Some patients were bitten for more than 1 year, hence the age ranges.

† , Persistence: if symptoms such as seal finger were present, how long did these persist and with what treatment.

### Bite treatment

Of the 22 cases of seal bites reported (Table 1), four cases were not treated, but some cleaning might have taken place during normal bathing of biologists. The remaining 18 cases were cleaned by flushing with saline, water or an antiseptic solution. Four of these cases were sutured to facilitate closure of wounds. Antibiotics were used prophylactically in seven cases. Six cases developed no signs of infection and the remaining case recovered within 4 days. Reported antibiotics used were amoxicillin+clavulanic acid or tetracyclines. The majority of wounds healed uneventfully. Only three cases (Table 1: cases 5, 7 and 13) showed symptoms of potential infection characterised by swelling, stiffness or pain. The remaining four cases (Table 1: cases 3, 4, 6 and 8) showed symptoms indicative of normal inflammatory response involved in wound healing and recovered without the use of antibiotics.

## Discussion

**FIGURE 2:**
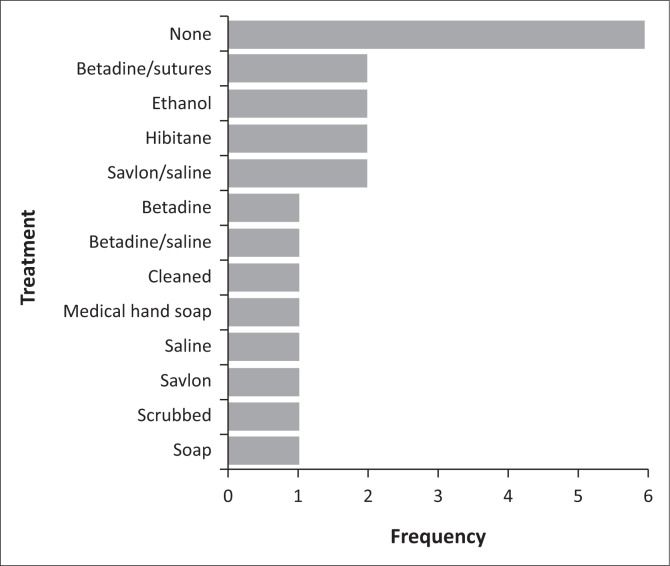
Initial treatment of seal bite cases at Marion Island, 1995–2012.

The use of at least 13 different wound treatment methods (Figure 2) amongst the 22 bite cases that we examined (Table 1) indicates a lack of knowledge of optimal in-field treatment of mammalian bite wounds.

All mammalian bite wounds are considered grossly contaminated (Ellis & Ellis 2014). Contamination is the presence of bacteria in the wound at concentrations that do not delay healing. Without timeous and appropriate treatment contamination may progress to infection, defined as the presence of replicating organisms in the wound with subsequent host injury (Edwards & Harding 2004), and may be characterised by fever, redness, swelling, pain, heat, loss of function and discharge from the wound (DeBoard et al. 2007; Edwards & Harding 2004; Patronek & Slavinski 2009; Velnar, Bailey & Smrkolj 2009) beyond the normal inflammatory phase. The wound healing process, which starts at the time of initial injury, can be arbitrarily divided into the following four main phases: (1) *Coagulation and haemostasis*. (2) *Inflammation*: the normal physiological response of the body to injury, clinically recognised in wounds by redness, swelling, pain, heat and loss of function. It establishes an immune barrier against invading micro-organisms, clears the wound of contaminants and foreign material and provides the injury site with the cells and chemical mediators needed for wound healing. It usually does not last for more than a few days (Broughton, Janis & Attinger 2006; Guo & DiPietro 2010; Reinke & Sorg 2012; Velnar et al. 2009). (3) *Proliferation*: it starts 3–10 days post-injury, covers the wound with granulation tissue, restores the vascular network, re-epithelialises the wound surface and decreases the surface area of the wound through contracture (Reinke & Sorg 2012). (4) *Wound remodelling*: connective tissue is realigned along tension lines and unneeded cells are removed (Reinke & Sorg 2012).

Following a seal bite, accepted bite wound management principles should be followed to optimise healing and prevent infection. As the most important step in optimising wound healing (Nicks et al. 2010), we recommend immediate (Patronek & Slavinski 2009) and thorough (Nicks et al. 2010) lavage of the wound with a physiological, sterile, non-cytotoxic solution such as saline or potable water (Fernandez & Griffiths 2008) until it is macroscopically clean to decrease contaminants and remove foreign material (Edwards & Harding 2004; Ellis & Ellis 2014; Kennedy, Stoll & Lauder 2015; Morgan & Palmer 2007; Owens & Wenke 2007; Patronek & Slavinski 2009; Smith, Walker & Brenchley 2003). Lavage can easily be performed in the field using a 20-mL syringe attached to a 21-G needle (Lam, Rastomjee & Dynan 2000) or 20-G catheter (Ellis & Ellis 2014). Surrounding skin should be cleaned (Ellis & Ellis 2014), dried and a sterile, non-adherent, semi-permeable, absorptive dressing must be applied to prevent further contamination of the wound environment, and to absorb wound exudate and promote wound healing (Abdelrahman & Newton 2011; DeBoard et al. 2007). Direct pressure can be applied over this dressing to stem any active bleeding (Edlich et al. 2010). Open wound management should be continued until the debridement phase is complete, followed by delayed primary or secondary wound closure (Edlich et al. 2010; Ellis & Ellis 2014; Jha, Khan & Siddiqui 2014; Morgan & Palmer 2007; Patronek and Slavinski 2009; Stevens et al. 2014). Prophylactic antibiotics are generally not indicated for ‘low risk’ bites (Morgan & Palmer 2007) but if deemed necessary, as in the case of deep puncture wounds of the hand, finger tendon sheaths or joints (Ellis & Ellis 2014; Jha et al. 2014; Kennedy et al. 2015), and in immune-compromised patients (Ellis & Ellis 2014), these should be directed against known pathogens of the biting animal’s oral flora and the victim’s skin (Patronek & Slavinski 2009). Antibiotics should be used as an adjunct to thorough wound washout as opposed to an alternative (Malahias et al. 2014). With signs of wound infection, microbial culture and sensitivity testing should be used for selection of appropriate antibiotics (Kennedy et al. 2015; Malahias et al. 2014; Nicks et al. 2010; Patronek & Slavinski 2009). Tetanus vaccination should be administered if the victim was immunised for more than five years prior to the bite (Ellis & Ellis 2014).

The higher incidence of bites on the fingers, hands and arms is expected because of the nature of the work being performed. However, it may also indicate the lack of use of appropriate protective clothing.

In spite of more than 40 years of seal research at Marion Island and thousands of contacts with three species of seal, no case of seal finger has been reported from the island. Our examination of 22 bite cases from the island provides little to no evidence of seal finger on the island. The stiffness experienced by the patient in case 13 (Table 1) might have been because of bruising and damage during the bite. Similarly, in spite of widespread sealing in the Antarctic and sub-Antarctic and extensive seal research activities (Basberg & Headland 2013; Laws 1993), few cases of possible seal finger were reported from the southern hemisphere (Cawthorn 1994; Liavaag 1940; Panagis et al. 1982). The two fur seal species occurring on Marion Island are from the same genus (*Arctocephalus*) as the fur seal species responsible for two of the aforementioned bite cases. Furthermore, *Mycoplasma phocicerebrale*, the putative causative agent of seal finger, has been isolated recently from Australian fur seals, *Arctocephalus pusillus doriferus* (Lynch et al. 2011). Handling southern elephant seal carcasses caused seal finger amongst sealers at South Georgia (Liavaag 1940) and a northern elephant seal, *Mirounga angustirostris*, bite has likely caused seal finger (Lewin, Knott & Lo 2004). Some cases of seal finger may have been diagnosed as erysipeloid (e.g. Hillenbrand 1953), but given the northern hemisphere provenance of most of the sealing vessels and crews in the Antarctic, it is very likely that Antarctic sealers would have been familiar with seal finger.

In 68.2% of bite cases, no antibiotics were used. In the remaining 31.8% of bite cases, at least two different antibiotics were used. Therefore, we conclude that the causative agent for seal finger was apparently not present during these bites or that lavaging most of them removed the organism.

## Summary and recommendations

**FIGURE 3:**
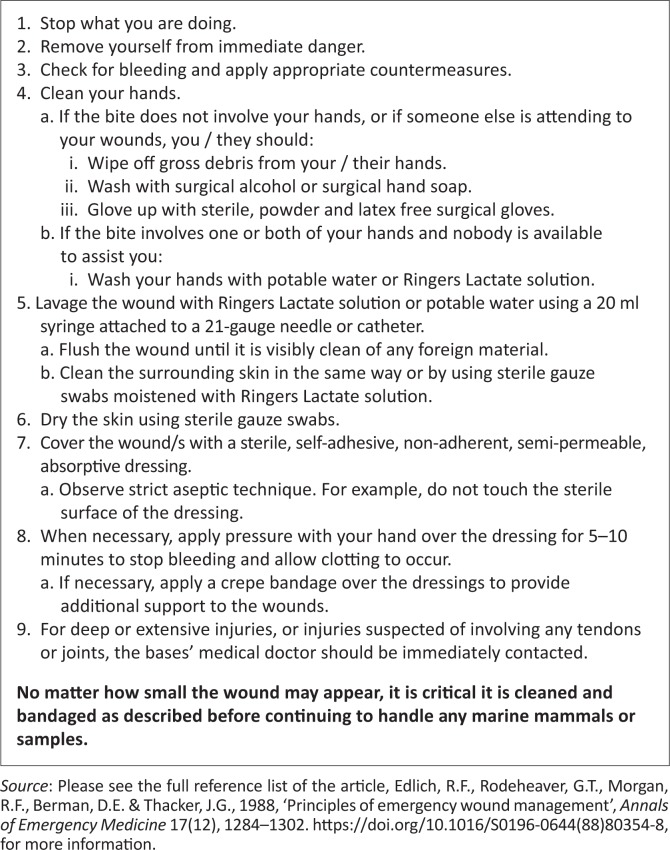
Recommended in-field treatment of seal bites on Marion Island to minimise wound contamination and infection with pathogenic organisms.

The large variety of wound treatments administered indicates a need for standardised training of personnel with currently accepted best medical practice (Figure 3) for the treatment of mammalian bites.

Firstly, we recommend that marine mammal biologists, veterinarians and medical staff at Marion Island and other Antarctic and sub-Antarctic research stations are to be better educated regarding seal finger and its treatment as well as in-field medical treatment of seal bites. Secondly, we recommend sealers must have immediate access to a 1-L bag of Ringer’s lactate or potable water with 20-mL syringe and 21-G needle or catheter for in-field irrigation of seal bites together with a few sterile adhesive, non-adherent, semi-permeable, absorptive wound dressings as the most appropriate initial treatment for seal bites (Figure 3). Tetracycline should be used when seal finger is suspected, but because of the similarity of signs with *Erysipelothrix* (Hillenbrand 1953), a penicillin product should be administered concurrently (Stevens et al. 2014). The absence of reported seal finger from the island suggests that the putative agent may not be present. Better reporting of bites and their treatments would facilitate further investigations of seal finger.
